# Supplementary material for: The Reduction in Gastric Atrophy after Helicobacter pylori Eradication Is Reduced by Treatment with Inhibitors of Gastric Acid Secretion
Source: Int J Mol Sci. 2019 Apr 18;20(8):1913. doi: 10.3390/ijms20081913 (PMC6515232; doi:10.3390/ijms20081913)
Supplement: Supplementary file 1 [file ijms-20-01913-s001.pdf]

Supplementary Table S1. Association between changes in atrophy score and CDX1 expression (N = 23)

Atrophy change	CDX1 expression, mean ± SD		P value
	**Initial assessment**	**Final assessment**	
Improvement			
Antrum (N = 12)	0.099 ± 0.106	0.034 ± 0.060	0.136
Corpus (N = 7)	0.093 ± 0.113	0.056 ± 0.072	0.564
Exacerbation			
Antrum (N = 1)	0.491	0.015	Not applicable
Corpus (N = 3)	0.009 ± 0.007	0.120 ± 0.123	0.258

Improvement and exacerbation were defined as a decrease and increase, respectively, in the updated Sydney system score between the first and final biopsies.

Supplementary Table S2. Association between changes in the atrophy score and CDX2 expression (N = 39).

Atrophy change	CDX2 expression, mean ± SD		P-value
	Initial assessment	Final assessment	
Improvement			
Antrum (N = 19)	0.071 ± 0.120	0.054 ± 0.160	0.636
Corpus (N = 12)	0.076 ± 0.142	0.090 ± 0.202	0.807
Exacerbation			
Antrum (N = 2)	0.316 ± 0.440	0.100 ± 0.041	0.637
Corpus (N = 6)	0.012 ± 0.009	0.102 ± 0.159	0.222

Improvement and exacerbation were defined as a decrease and increase, respectively, in the updated Sydney system score between the first and final biopsies.

Supplementary Table S3. Association between changes in the metaplasia score and incomplete metaplasia (N = 63).

Metaplasia change	Case with incomplete metaplasia		P-value
	Initial assessment	Final assessment	
Improvement			
Antrum (N = 25)	8 (32.00%)	3 (12.00 %)	0.215
Corpus (N = 11)	5 (45.45%)	4 (36.36 %)	1.000
Exacerbation			
Antrum (N = 21)	4 (19.05%)	11 (52.38%)	0.090
Corpus (N = 6)	3 (50.00%)	1 (16.67%)	1.000

Supplementary Table S4. GSRS symptom scores 3 months after eradication (N = 65)

**GSRS score item**	**GSRS score**		**P-value**
	**Initial assessment**	**Final assessment**	
GSRS1: Have you been bothered by PAIN OR DISCOMFORT IN YOUR UPPER ABDOMEN OR THE PIT OF YOUR STOMACH during the past week?	1.673 ± 1.167	1.354 ± 0.758	**0.004**
GSRS2: Have you been bothered by HEARTBURN during the past week?	1.635 ± 1.048	1.291 ± 0.582	**0.018**
GSRS3: Have you been bothered by ACID REFLUX during the past week?	1.519 ± 0.918	1.400 ± 0.707	0.376
GSRS4: Have you been bothered by HUNGER PAINS in the stomach during the past week?	1.481 ± 0.779	1.208 ± 0.504	**0.002**
GSRS5: Have you been bothered by NAUSEA during the past week?	1.423 ± 1.144	1.271 ± 0.707	0.236
GSRS6: Have you been bothered by RUMBLING in your stomach during the past week?	1.500 ± 0.804	1.979 ± 1.280	**0.007**
GSRS7: Has your stomach felt BLOATED during the past week?	1.712 ± 1.177	1.458 ± 0.651	**0.025**
GSRS8: Have you been bothered by	1.481 ± 1.038	1.480 ± 0.799	0.490

BURPING during the past week?

GSRS, gastrointestinal related gastrointestinal symptom rating scale

Bold, statistical significance.

Supplementary Table 5. GSRS symptom scores for all observational periods after eradication during all follow-up periods (N = 52)

GSRS score item	GSRS score		P-value
	Pre-eradication	Post-eradication	
GSRS1: Have you been bothered by PAIN OR DISCOMFORT IN YOUR UPPER ABDOMEN OR THE PIT OF YOUR STOMACH during the past week?	1.600 ± 1.087	1.577 ± 1.273	0.501
GSRS2: Have you been bothered by HEARTBURN during the past week?	1.538 ± 0.969	1.750 ± 1.100	0.572
GSRS3: Have you been bothered by ACID REFLUX during the past week?	1.462 ± 0.849	1.519 ± 0.851	1.000
GSRS4: Have you been bothered by HUNGER PAINS in the stomach during the past week?	1.431 ± 0.749	1.442 ± 0.873	0.542
GSRS5: Have you been bothered by NAUSEA during the past week?	1.369 ± 1.039	1.481 ± 0.980	0.920
GSRS6: Have you been bothered by RUMBLING in your stomach during the past week?	1.538 ± 0.885	1.827 ± 1.133	0.236
GSRS7: Has your stomach felt BLOATED during the past week?	1.692 ± 1.131	1.673 ± 0.833	0.442
GSRS8: Have you been bothered by	1.554 ± 1.061	1.673 ± 1.184	0.646

BURPING during the past week?

GSRS, gastrointestinal related gastrointestinal symptom rating scale
